# Validation of the relevant outcome scale for Alzheimer's disease: a novel multidomain assessment for daily medical practice

**DOI:** 10.1186/alzrt89

**Published:** 2011-09-14

**Authors:** Vjera A Holthoff, Steven Ferris, Ralf Ihl, Philippe Robert, Bengt Winblad, Serge Gauthier, Kati Sternberg, Frank Tennigkeit

**Affiliations:** 1 Old Age Psychiatry and Cognitive Neuropsychiatry, University Hospital Carl Gustav Carus, University of Technology, Fetscherstraße 74, Dresden 01307, Germany; 2 DZNE, German Center for Neurodegenerative Diseases, Dresden, Germany; 3 Alzheimer's Disease Center, New York University Langone Medical Center, 550 First Avenue, New York, NY 10016, USA; 4 Clinic of Geriatric Psychiatry and Psychotherapy, Alexian Krefeld GmbH, Diessemer Bruch 81, Krefeld 47805, Germany; 5 University of Düsseldorf, Universitätsstraße 1, 40225 Düsseldorf, Germany; 6 Memory Center EA CoBTek, Université de Nice Sophia Antipolis, Hôpital de Cimiez, 4 av Victoria 06000, Nice, France; 7 Karolinska Institutet Alzheimer Disease Research Center, Novum 5th Floor, SE-141 86 Stockholm, Sweden; 8 Alzheimer's Disease Research Unit, McGill Centre for Studies in Aging, 6825 LaSalle Boulevard, Verdun, Quebec H4H 1R3, Canada; 9 Merz Pharmaceuticals GmbH, Eckenheimer Landstraße 100, Frankfurt 60318, Germany; 10 AC Immune SA, PSE Building B - EPFL, CH-1015 Lausanne, Switzerland

## Abstract

**Introduction:**

The Relevant Outcome Scale for Alzheimer's Disease (ROSA) is a new observer rating instrument recently developed for routine medical practice. The validity and reliability of ROSA as well as sensitivity to changes due to intervention were examined in an open-label, single-arm, multicenter clinical study in patients with Alzheimer's disease (AD).

**Methods:**

The study enrolled 471 patients with a diagnosis of AD consistent with the criteria of the National Institute of Neurological and Communicative Disease and Stroke/Alzheimer's Disease and Related Disorders Association or with the Diagnostic and Statistical Manual Disorders criteria for dementia of Alzheimer's type. Following assessments of the ROSA and other standard assessments (Alzheimer's Disease Assessment Scale - cognitive subscale, Severe Impairment Battery, Neuropsychiatric Inventory, and Disability Assessment for Dementia), patients were treated with memantine for 12 weeks. Factor analysis of the baseline ROSA total scores was performed based on the principal components method using the varimax orthogonal rotational procedure. The psychometric analyses of the ROSA included internal consistency, test-retest reliability, inter-rater reliability, construct validity, and responsiveness to changes over time.

**Results:**

All items showed adequate factor loadings and were retained in the final ROSA as Factor 1 (all items related to cognition, communication, function, quality of life and caregiver burden) and Factor 2 (all behavior items). The ROSA demonstrated high internal consistency (Cronbach's α = 0.93), test-retest reliability (intraclass correlation coefficient = 0.93), and inter-rater reliability (intraclass correlation coefficient = 0.91). The correlation coefficients between the ROSA and each of the validated scales ranged between 0.4 and 0.7, confirming the ROSA construct validity. Nonsubstantial floor and ceiling effects were found in middle and late disease stages, whereas a small ceiling effect was observed in the early stage. The ROSA responsiveness to change was high (responsiveness index ≥0.8) for all severity stages.

**Conclusions:**

The ROSA is a valid and reliable instrument to aid medical practitioners in sensitively assessing AD-relevant symptoms over time in their clinical practice.

## Introduction

The Relevant Outcome Scale for Alzheimer's Disease (ROSA) is a novel observer rating instrument recently developed for daily clinical practice to allow physicians and other medical practitioners with expertise in the management of patients with dementia (for example, psychologists, trained raters) to determine the severity of relevant symptoms in Alzheimer's disease (AD) and to document disease progression and therapy effects over time. The need for an instrument such as the ROSA was identified after a comprehensive literature survey of existing AD rating scales widely used in clinical practice, and after extensive discussions with medical practitioners and caregiver experts [1]. The following requirements were determined for an ideal practical scale: easy and quick administration; high reliability and validity for AD; multidomain assessment of cognition, activities of daily living, behavior, communication, quality of life, and caregiver burden; relevance for all AD severity stages; suitability for long-term monitoring disease progression; and high sensitivity to treatment effects [1]. Given these scale characteristics, the ROSA was developed as a clinician assessment scale, including 16 items grouped into six dimensions (cognition, communication, behavior, function, quality of life, and caregiver burden). The items were selected by a standard content validity approach based on literature data and critical judgment of experts and caregivers on the most practice-relevant assessment criteria for global clinical evaluation of disease progression [1]. Altogether, the ROSA uses 14 items for assessment of the actual clinical status of a patient in terms of patient impairment and behavior, and two items for evaluation of patient quality of life and caregiver burden.

Three AD severity stages are designated in the ROSA - early, middle and late. A number of scales and rating systems are presently used for AD staging in clinical practice and research [2-5]; however, the timing and course of an individual disease progression can vary greatly from one patient to another. Some broadly utilized staging instruments, such as the Global Deterioration Scale (GDS) or the Clinical Dementia Rating Sum of Boxes [2,3], may be used to determine the patient's disease stage prior to the ROSA administration. A concise description of the three severity stages within the ROSA is provided in the ROSA manual, which is a part of the instrument. The stages are based on cognitive, functional, and behavioral disease symptoms of a patient and correspond to the widely used concepts of mild, moderate and severe AD. Briefly, in the early stage, cognitive deficits include, for example, word-finding and name-finding difficulties, memory complaints for recently learned information, or regularly misplacing valuable objects. Patients may reveal troubles in planning, initiating, and performing in social settings; close relatives, friends, or colleagues also start noticing cognitive deficits in patients, which may not be apparent to others. In the middle stage, forgetfulness for recent events and autobiographical memories is increased, performance in complex tasks is worse, and depressed mood, withdrawal in a social situation, or other behavioral changes may become noticeable. Patients demonstrate difficulties in activities of daily living and orientation. In the late stage, patients lose awareness of recent events and of personal history, and the need for help in activities of daily living is high. The ability to respond to the environment as well as orientation in space and time is severely impaired. Personality and behavioral changes may also take place.

The rater has to appraise the patient's disease stage (early, middle, or late) before applying the ROSA evaluation. A condition for the use of the ROSA is that the rater - who can be the treating clinician or psychologist in clinical settings, or also the nurse involved in medical care - is familiar with the necessary background information on the patient so that it can be incorporated as part of the assessment. The staging must be based on the comprehensive clinical impression about the patient.

The evaluation with the ROSA is made with the aid of scenarios (events or situations). In the ROSA, everyday scenarios are described for Items 1 to 14. The scenario of an item remains the same for each severity stage, but it is supplemented by three examples that describe the capacities/behavior of the patient at each of the three severity stages. Should a scenario of a particular ROSA item not be applicable to an individual patient, the ROSA provides the possibility of using alternative scenarios taking into account eventual cultural or gender differences. A few examples of alternative scenarios are given in the ROSA manual to illustrate this possibility. In such cases, the rater is required to note the alternative scenarios used for the first assessment with the ROSA and to apply these scenarios in any subsequent ratings.

The ROSA is administered in interview form. The interview partner is usually the primary caregiver. In the early stage of disease, the patient alone can also be the interview partner. During the interview, the rater asks questions based on the scenarios defined in the ROSA and makes an estimate of each item on a numerical scale of 0 to 10. Higher scores indicate better patient abilities/quality of life and less caregiver burden. The final estimate is the ROSA total score given by the sum of all 16 single-item scores (range 0 to 160). The range of the total score is the same for all severity stages, higher scores indicating less impairment and burden. Complete instructions on the ROSA use in daily clinical practice are provided with the ROSA manual. Both the ROSA and the ROSA manual can be obtained upon request from Merz Pharmaceuticals GmbH (Frankfurt am Main, Germany) and will be free of charge for noncommercial use.

In the present paper, we present the structure and psychometric properties of the ROSA that were analyzed in an open-label, single-arm, multicenter clinical trial in patients with AD of all severity stages. The primary goal of the study was to investigate the validity and reliability of the newly developed instrument, the ROSA, as a unique tool for assessment of AD progression in daily clinical practice. The ability of the ROSA to measure changes within persons over time, the so-called responsiveness, was also tested based on the study results and reported here.

## Materials and methods

### Study design and population

This open-label, multicenter, single-arm clinical study was carried out in 32 primary care centers and 15 outpatient clinics in Germany and Austria. The raters were professionals familiar with the patient's medical history and experienced in disease staging, progression, and dementia care: treating clinicians and psychologists in clinical settings as well as nurses involved in medical care. All raters underwent training for administration of the ROSA prior to the study start, participating in a workshop of 5 hours. Patients with a diagnosis of AD consistent with the National Institute of Neurological and Communicative Disease and Stroke/Alzheimer's Disease and Related Disorders Association criteria or with the Diagnostic and Statistical Manual Disorders IV-TR criteria for dementia of Alzheimer's type were enrolled. Patients taking memantine or any contraindicated medications (for example, anticonvulsants, anti-Parkinson drugs, or other N-methyl-D-aspartate receptor blockers) within the past 6 months were not eligible, but those on a stable dosage of cholinesterase inhibitors for at least 2 months prior to study enrollment were eligible. Patients with clinically significant, unstable central nervous system or psychiatric disease other than AD, including bipolar or unipolar depression, were also not eligible. The majority of the patients had a knowledgeable caregiver participating in the study. The study was conducted in accordance with the principles of good clinical practice and the World Medical Association Declaration of Helsinki 1964 and its amendments and subsequent clarifications. The relevant local ethics committees approved the study and written informed consent was obtained from patients or patients' legally acceptable representatives.

Out of 487 patients screened, 471 were eligible for inclusion into the study. Following baseline assessments, 451 patients were treated with memantine for 12 weeks (20 mg/day once daily). For statistical analysis, the population was defined as: the safety evaluation set, including all patients who took at least one dose of study medication (451 patients, 95.8%); and the full analysis set (FAS), consisting of all patients of the safety evaluation set for whom ROSA data were available from screening to the study end (397 patients, 84.3%). Most patients had a caregiver to accompany the patient to all study visits and provide study-relevant information about the patient.

### Measures

Assessments with the ROSA were performed at screening and at baseline visits by the same rater for evaluation of test-retest reliability. For initial AD staging, the GDS was used at screening. At baseline, patients/caregivers were asked to complete the Alzheimer's Disease Assessment Scale - cognitive subscale (ADAS-cog) [6], the Severe Impairment Battery (SIB) [7], the Neuropsychiatric Inventory (NPI) [8], the Disability Assessment for Dementia (DAD) [9], and the Mini-Mental State Estimation (MMSE) [5]. The ADAS-cog was administered only to patients in the early and middle AD severity stages, and the SIB to those in the middle and late AD severity stages. The scores obtained in Questions 1, 39, and 40 of the SIB did not enter analyses. The time for scale administrations was recorded by the clinician.

### Reliability analyses

To assess internal consistency of the ROSA, the Cronbach α coefficient [10] of the ROSA construct was calculated from baseline data. As an indicator of the scale's reliability, the measurement error (*S_ε_*) was evaluated according to the formula:

$$S_{\varepsilon }=S_{x}\sqrt{1-Cronbach^{\prime }s\cdot \alpha },$$

where *S_x _*is the standard deviation of the ROSA total score at baseline. Inter-rater reliability was evaluated based on videotaped ROSA administrations showing a physician applying the ROSA to the caregiver of a patient with early, middle, or late AD. Prior to the assessment, raters were given clinical information on the patients' history, such as biographical facts, information on disease manifestation, disease duration, treatment, and psychometric tests (GDS and MMSE). The intraclass correlation coefficient (ICC, Shrout-Fleiss reliability single score) was computed based on assessments from 61 raters; that is, investigators from all 47 dementia care centers who participated in the ROSA validation study. Test-retest reliability was determined by two administration of the ROSA by the same rater within a period of up to 10 days prior to any treatment. As quantitative measures of the reliability, Bravais-Pearson and ICC coefficients were used.

### Validity analyses

To evaluate the construct validity of the ROSA, a factor analysis based on the principal components method using an orthogonal rotational procedure (Varimax) was applied on baseline ROSA data. Initial factors were selected on the basis of eigenvalues ≥1 (Kaiser-Guttmann criterion). A threshold of ≥0.4 for single-item loadings of each identified factor was predefined to decide which items should remain in ROSA. Items with a loading > 0.4 for more than one identified factor were considered a part of the factor in which it has the highest loading. In addition, a scree test was applied as an alternative criterion for the number of extracted factors.

The ROSA's concurrent validity was tested by comparison with the ADAS-cog, SIB, DAD, and NPI, and was assessed by means of the Bravais-Pearson correlation coefficient. A range of 0.4 to 0.7 for the correlation coefficients between the ROSA and each of the validated scales was predefined as acceptable to confirm the ROSA construct validity.

### Responsiveness analyses

The responsiveness of the ROSA to change after 12-week treatment with memantine, 20 mg per day, was determined by means of the responsiveness index (RI) and Cohen's *d *effect size [11,12]. The RI was calculated according to the formula:

$$RI_{\left(x\right)}=\frac{{\bar{X}}_{+}}{S_{x_{0}}}$$

where ${\bar{X}}_{+}$ is the arithmetic mean of pre-post differences (week 12 to baseline) of the ROSA scores for all patients with positive change in the total score (pre-post difference > 0), and $S_{x_{0}}$ is the standard deviation of pre-post differences of the ROSA score for all patients with negative or no change in the total score (pre-post difference ≤0). The analysis was performed using pooled and severity-stage datasets. Only patients with nonmissing values and retaining the severity stage throughout the study were considered for evaluation. The Cohen's *d *value was calculated as the difference between mean ROSA total scores at week 12 and baseline, divided by the standard deviation of the pooled dataset. According to Cohen [12], values of 0.20 to 0.49 indicate a small effect size, values of 0.50 to 0.79 a moderate effect size, and values ≥0.8 a large effect size.

All analyses were performed using SAS software, version 9.1.3 (SAS, Inc., Cary, NC, USA).

## Results

The ROSA validity and reliability were analyzed using the safety evaluation set as well as the FAS baseline study data, both resulting in the same factor structure of the ROSA and similar psychometric characteristics. In the present article, only results obtained from the FAS analysis will be presented because, besides validity and reliability of the ROSA, the FAS analysis also provides an estimate of the ROSA responsiveness; that is, the ability of the scale to detect changes over time due to a treatment, which is an essential psychometric property of all evaluative instruments.

Table 1 presents the baseline demographic characteristics of the FAS population, overall and by disease severity stages. All patient groups were comparable with regard to age and gender proportions. Overall, the average age of the patients was 75.7 years and women constituted 56.7% of the FAS population. The MMSE total score distribution across the AD severity groups showed a decrease in the mean MMSE score from the early (23.3 ± 4.2) to the late (12.5 ± 5.5) disease stages. The mean total scores of the other scales administered at baseline (ADAS-cog, SIB, DAD, and NPI) also demonstrated an increasing level of relevant impairment from early to late disease severity stages (Table 1).

**Table 1:** Demographic data at baseline

	Early stage	Middle stage	Late stage	Overall
Age (years)	74.1 (7.91)	76.3 (7.12)	78.3 (6.07)	75.7 (7.4)
Gender, female	84 (52.2%)	100 (57.1%)	41 (67.2%)	225 (56.7%)
White	161 (100.0%)	175 (100.0%)	61 (100.0%)	397 (100%)
Weight (kg)	74.0 (13.7)	72.0 (15.1)	67.6 (11.5)	72.1 (14.1)
Height (cm)	167.0 (8.9)	165.1 (9.1)	164.0 (8.1)	165.7 (8.89)
MMSE score	23.3 (4.2)	18.6 (5.2)	12.5 (5.5)	19.6 (6.1)
ADAS-cog score	16.3 (6.4)	23.4 (8.4)	-	20.0 (8.3)
SIB score^a^	-	79.1 (11.3)	62.6 (20.3)	74.7 (16.0)
DAD score	33.3 (6.8)	24.7 (9.4)	13.8 (8.3)	26.4 (10.6)
NPI score	9.7 (9.6)	14.1 (11.7)	22.2 (14.4)	13.9 (12.3)

Data presented as mean (standard deviation) or number (%) of patients (full analysis set). ADAS-cog, Alzheimer's Disease Assessment Scale - cognitive subscale; DAD, Disability Assessment for Dementia; MMSE, Mini-Mental State Estimation; NPI, Neuropsychiatric Inventory; SIB, Severe Impairment Battery.^a^Questions 1, 39, and 40 were omitted in this study.

A requirement for using the ROSA in the clinical study was that the clinician is familiar with the necessary background information on the patient. All study investigators were trained that the disease staging prior to the ROSA administration should be most of all based on the comprehensive clinical impression about the patient. In addition, a structured global staging instrument such as the GDS was used in the study to determine the severity stage of the patient for the use of the ROSA. The GDS was applied at screening before the ROSA administration in order to guide and standardize the initial severity staging in the ROSA.

### Construct validity

The Kaiser-Guttmann criterion as well as the scree test produced two ROSA factors as follows: Factor 1, including Items 1 to 6 and Items 12 to 16; and Factor 2, including Items 7 to 11 (Table 2). All 16 ROSA items showed adequate factor loadings (≥0.4) and therefore were retained in the final ROSA. Only Item 15, assessing patient's quality of life, showed a loading higher than the defined threshold of 0.4 in both Factor 1 and Factor 2 (a loading of 0.4063 in Factor 2). The resulting Factor 1 comprises the AD-relevant symptom domains of cognition, communication, and function/activity of daily living, as well as patient quality of life and caregiver burden. Factor 2 comprises all of the items relevant for the assessment of behavior. Both factors showed eigenvalues higher than 1, explaining 57% of the total variance.

**Table 2:** Factor analysis of the ROSA (baseline data, FAS)

ROSA item		Symptom domain	Factor	Loading
1	The patient is able to remember events from a long time ago.	Cognition	Factor 1	0.6857
2	The patient is able to remember events of the recent past.	Cognition		0.7448
3	The patient is able to plan and carry out complex procedures.	Cognition		0.8526
4	The patient is able to make himself understood.	Communication		0.7138
5	The patient is able to communicate.	Communication		0.7966
6	The patient shows social competence.	Communication		0.7289
12	The patient is competent at everyday tasks.	Function/ADL		0.7791
13	The patient is attentive, shows interest in his surroundings.	Function/ADL		0.7284
14	The patient is independent.	Function/ADL		0.7849
15	The patient's quality of life is (very good/very poor).	Quality of life		0.4509
16	The burden for the caregiver is (very small/very large).	Caregiver burden		0.6183
7	The patient behaves aggressively.	Behavior	Factor 2	0.7309
8	The patient is restless.	Behavior		0.6248
9	The patient shows behavioral changes due to delusions.	Behavior		0.7397
10	The patient is insecure.	Behavior		0.5014
11	The patient's behavior is cooperative.	Behavior		0.6243

ADL, activity of daily living.

The Bravais-Pearson correlation coefficient was examined by comparing the ROSA baseline data with baseline data for the ADAS-cog, SIB, NPI, and DAD (Table 3). The ROSA/ADAS-cog correlation was assessed for early and middle disease stages only, while the ROSA/SIB correlation was assessed for middle and late disease stages. The values of the correlation coefficients varied within the predefined range for all scales, confirming an adequate correlation between the ROSA and each of the other scales, and thus supporting the construct validity of the ROSA.

**Table 3:** Correlation of ROSA total scores with ADAS-cog, SIB, NPI, and DAD scores

	*n*	Bravais-Pearson coefficient
ADAS-cog	332	-0.4724
Severe Impairment Battery (SIB)	227	0.5000
Neuropsychiatric Inventory (NPI)	391	-0.5383
Disability Assessment for Dementia (DAD)	384	0.7060

ADAS-cog, Alzheimer's Disease Assessment Scale - cognitive subscale; *n*, total number of patients (full analysis set); ROSA, Relevant Outcome Scale for Alzheimer's Disease. Missing item value imputation was applied for the ROSA, SIB, ADAS-cog, and NPI. Completely missing values for a scale were not replaced. Evaluations included only patients with available data for both correlated scales.

### Internal consistency

The resulting value of Cronbach's α was 0.9279, indicating high internal consistency of the ROSA. A measurement error of 7.66 points was calculated on the basis of the standard deviation and Cronbach's α as an indicator of the scale's reliability.

### Inter-rater reliability

Data were obtained from independent assessments by 61 raters of three videotaped ROSA administrations, one for each AD severity stage. Each rater provided assessments of at least two videos, thus resulting in 122 assessments (early stage, 42 assessments; middle stage, 40 assessments; late stage, 40 assessments) used for analysis. Given that values of the ICC coefficient ≥0.8 indicate a high intra-class correlation, the resulting coefficient of 0.9056 demonstrates a high inter-rater reliability for the scale.

### Test-retest reliability

**Figure 1:**
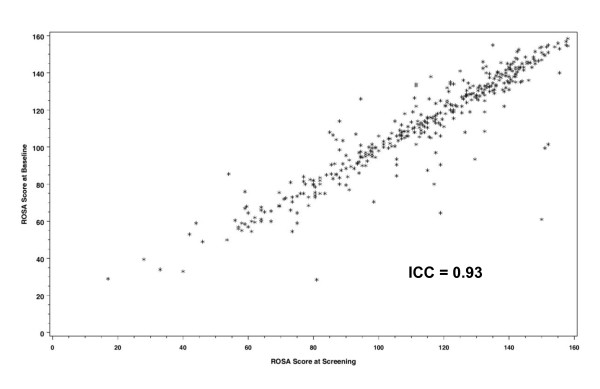
**Test-retest reliability**. Correlation between the Relevant Outcome Scale for Alzheimer's Disease (ROSA) total scores at screening and baseline. ICC, intraclass correlation coefficient.

Figure 1 shows the distribution of the ROSA total scores at screening versus baseline. Only patients with nonmissing values for both assessments were used for the test-retest reliability analysis (382 patients of the FAS). The time between the initial ROSA administration and the ROSA retest was 6.6 ± 1.4 days (range of 1 to 10 days between screening and baseline visits). During this period the patients were not treated with memantine. Given the short time between the test and the retest and the missing influence of treatment, no systematic changes or learning effects were expected to occur. The correlation estimated by both the Bravais-Pearson coefficient and the ICC (0.9309 and 0.9301, respectively) were higher than 0.7, indicating an excellent test-retest reproducibility for the ROSA.

### Responsiveness

To investigate the extent to which the ROSA could measure relevant changes due to a treatment in patients with early, middle, and late AD, the responsiveness of the ROSA to change was assessed comparing baseline ROSA data with the ROSA total scores after 12-week treatment with memantine. The RI values determined by disease stage were 0.81 for early stage, 1.54 for middle stage, and 1.70 for late stage. For the overall study population, the RI was 1.25. Based on the defined ranges according to Cohen [11], RI values ≥0.8 are considered to demonstrate a large sensitivity of ROSA to treatment effects. The ROSA therefore appeared to be sensitive to measure relevant changes due to treatment in all study populations (overall and severity-stage groups). The Cohen's *d *value was additionally estimated to appraise the magnitude of the treatment effect measured with the ROSA at study end. The results indicated a relatively small effect size of memantine in patients in middle stages (Cohen's *d *= 0.2104) and late stages (Cohen's *d *= 0.2232). In early AD, the effect size of memantine based on Cohen's *d *value (0.0340) was negligible.

### Floor and ceiling effects

**Figure 2:**
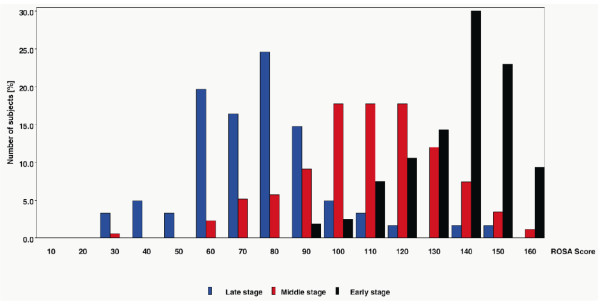
**Distribution of ROSA total scores at baseline in early, middle, and late disease stages**. ROSA, Relevant Outcome Scale for Alzheimer's Disease.

Possible floor and ceiling effects for each disease stage were analyzed based on the ROSA total score distribution at baseline (Figure 2). The results demonstrated that less than 5% of the patients in middle and late stages had scores close to the minimum and maximum ROSA total score, indicating no substantial floor/ceiling effects for this population. In the early stage, 9.3% patients had ROSA scores higher than 150, pointing to a small ceiling effect in early AD.

### Administration time

Table 4 provides an overview of the time needed for completion of ROSA and the other widely used and validated scales applied in the study. For all scales, the time for administration at baseline and at week 12 remained constant by severity stage and overall (Table 4). The mean value for ROSA completion ranged from 13 to 15 minutes for all stages. For comparison, the assessment of cognitive impairment with ADAS-cog or SIB scales took about 25 minutes, whereas 13 to 18 minutes were needed for NPI completion, and 10 to 12 minutes for the DAD.

**Table 4:** Time for administration of the ROSA and four other scales

Administration		Time (minutes)			
		Early stage	Middle stage	Late stage	Overall
ROSA	BL	13.6 (7.27)	15.5 (6.72)	14.4 (5.50)	14.5 (6.82)
	W12	13.2 (5.48)	15.3 (6.54)	14.3 (4.79)	14.3 (5.93)
ADAS-cog	BL	24.7 (7.39)	25.2 (7.51)	-	25.2 (7.51)
	W12	22.7 (7.45)	25.7 (8.06)	-	24.3 (7.89)
SIB	BL	-	24.8 (9.08)	29.0 (10.21)	26.3 (9.89)
	W12	-	24.5 (10.20)	26.4 (11.02)	25.4 (10.58)
NPI	BL	14.3 (6.60)	15.9 (11.87)	18.2 (7.81)	15.6 (9.54)
	W12	13.7 (7.20)	13.5 (6.46)	17.0 (8.40)	14.1 (7.19)
DAD	BL	10.8 (6.20)	11.5 (5.28)	12.4 (5.89)	11.4 (5.77)
	W12	9.9 (4.66)	11.3 (4.84)	11.6 (6.00)	10.8 (5.00)

Data presented as mean (standard deviation). ADAS-cog, Alzheimer's Disease Assessment Scale - cognitive subscale; BL, baseline; DAD, Disability Assessment for Dementia; NPI, Neuropsychiatric Inventory; ROSA, Relevant Outcome Scale for Alzheimer's Disease; SIB, Severe Impairment Battery; W12, week 12 after baseline.

## Discussion

Cognitive decline is the cardinal symptom in AD; however, noncognitive symptoms as well as patient quality of life and caregiver burden are also clinically relevant targets for treatment of AD [13]. Presumably, a set of rating scales would be necessary for use in daily medical practice, as in clinical trials of pharmacological and nonpharmacological interventions, to assess multiple AD-relevant domains. Assessment is therefore not only time consuming but current scales are not always applicable to all dementia stages and fail to measure therapy effects reliably [1]. A need for more sensitive instruments to detect AD symptom progression over time has been acknowledged [14,15].

The ROSA is a novel and unique multidomain assessment scale in AD, which has been designed for the need of daily clinical practice to assess disease progression over time and to measure dementia treatment effects. In the present article, we report the ROSA factorial structure based on clinical study data and discuss the psychometric characteristics of the scale, demonstrating that the ROSA is a valid, reliable and time-efficient observer-rating instrument to aid medical practitioners in sensitively assessing changes in AD symptoms over time.

The ROSA items cover a broad spectrum of AD symptoms, including cognitive impairment (Items 1 to 3), communication and social interaction abilities (Items 4 to 6), behavioral symptoms (Items 7 to 11), and symptoms relevant to activities of daily living (Items 12 to 14). Items 15 and 16 provide an overall evaluation of the patient's quality of life and the caregiver's burden, respectively. As demonstrated by the factor analysis, all items initially included in the ROSA were retained in the final ROSA clustered into two factors. The item assessing patient's quality of life (Item 15) showed the lowest loading within Factor 1, which indicates a rather weak relationship with the items comprising cognitive and functional AD symptoms in the ROSA. On the other hand, Item 15 showed a factor loading higher than the defined threshold of 0.4 also within Factor 2, implying that patient quality of life shows a similar relationship with all behavior items within the ROSA. This confirms the complexity of a patient's quality of life rating and the influence of a number of factors on the quality of life, including cognitive dysfunction, functional disabilities, and neuropsychiatric symptoms, as recently shown in a longitudinal study investigating the change in proxy-rated quality of life in a large cohort of home-living patients with AD [16]. The authors clearly demonstrated that measures of quality of life seem to comprise different functions than typical clinical variables such as cognitive and noncognitive dysfunction and activity of daily living [16]. It is also interesting that Item 13, describing the interest of the patient for his/her surroundings, was included in the ROSA factor comprising cognitive and functional/activity of daily living items (Factor 1). Given that this symptom is usually considered a core feature of apathy, its non-inclusion in the ROSA behavioral factor allows it to be focused only on the most disruptive symptoms.

High internal consistency and test-retest reliability of the final ROSA were shown, pointing to a high reproducibility of the ROSA results. The content validity analyses demonstrated a good correlation between the ROSA and each of the validated scales (ADAS-cog, SIB, NPI, and DAD) used as standard measures for the assessment of AD-relevant symptoms evaluated with the ROSA. In the current study, the ROSA was always applied prior to any other scale used in order to prevent a possible influence on the ROSA by the other scales. A putative limitation of the inter-rater reliability analysis applied in this study was that ROSA assessment was restricted to assessing the patient on video and to having clinical information on the patient only available in a written report. The results, however, showed good inter-rater reliability for the ROSA. A more direct approach for assessing inter-rater reliability required personal interviews by 61 raters in Germany and Austria, which was not feasible.

The ROSA responsiveness - that is, the ability of the ROSA to detect changes over time due to an intervention - was tested by two widely used criteria: the RI and Cohen's *d *coefficient. The RI provided an estimate of the ability of the ROSA to discriminate between patients who have changed due to treatment and those who have not benefited by the treatment. In turn, Cohen's *d *coefficient, which provides an estimate of the effect size due to an intervention, was analyzed to demonstrate the ability of the ROSA to detect any change over time due to memantine treatment. Given the differences in the responsiveness statistics of these two parameters, it is not surprising that the values of RI and Cohen's *d *differ greatly. One could expect that the RI values are higher than Cohen's *d *values because the RI numerator increases with exclusion of change scores from nonimproved patients, and the respective denominator becomes smaller with the inclusion of the standard deviation of change only for stable patients.

Altogether, the results indicate that the ROSA can sensitively measure relevant changes in all disease severity stages (RI ≥0.8 in all stages) and can reliably analyze the effect size of drug treatments. The latter was supported by the data for the ROSA estimate of the effect size due to treatment with memantine, which was shown to be within the known range of the memantine efficacy in moderate and severe AD stages [17]. Subsequent clinical experience with the ROSA, as well as its further use in clinical studies, would be necessary to permit an accurate assessment of the change in the total score that constitutes a clinically important difference, and thus to enable more accurate assessment of the ROSA responsiveness.

All clinicians taking part in the study were experienced professionals in AD diagnosis and treatment. Prior to initiating the study, all raters (physicians, psychologists and nurses) were trained in the application of the ROSA and the other AD scales.

With the ROSA training, the raters were instructed how to estimate a patient's disease severity prior to the ROSA administration and how to use the relevant examples of the scenarios within the ROSA. The raters were informed that the initial staging is an essential prerequisite for the ROSA administration; it establishes the assessment framework in the ROSA - that is, which example of a scenario (early, middle, or late) should be used for the assessment - and it ensures that, when patients' clinical symptoms are reassessed over time, any changes in the severity stage could be detected by the rater prior to the reassessment with the ROSA. Also, raters were instructed to be careful when applying modified scenarios; that is, to document any change of an item scenario and use the modified scenario again at the ROSA follow-up assessment. A video example of the ROSA administration was shown to demonstrate the use of the scenarios within the ROSA and the possibility to adjust an item scenario to the individual patient when needed.

For 95% of the study patients, the same clinician performed the assessments with the ROSA. The ROSA was scored on the basis of information obtained from the clinician interview and information available on the patient's medical history, clinical data, and previous encounters of the clinician. The interview partner was in most cases the primary caregiver who regularly sees the patient, thus being able to answer the questions on the basis of personal interaction with the patient during the preceding week.

The possibility to cover broad AD severity stages is a substantial advantage of the ROSA in daily clinical practice. One should, however, consider that the ratings are done for each stage using the same scoring range, while the scenarios used for each disease stage represent increasing levels of impairment. The ROSA total scores can thus be directly compared only within a patient or between patient groups of one and the same disease severity stage. This is a certain limitation in using the ROSA assessment in clinical trials for comparison of patient groups with different disease severity stages. Patients who change their severity stage over time may have to be excluded from statistical analyses in clinical trials; however, the individual results of the ROSA survey can still be compared relative to one another. Any change in the severity stage of a patient could be used as a global value of the AD progression and effects of an intervention. Besides, the same scoring range for all disease stages does not limit the use of the ROSA in routine medical practice where a single patient is assessed and comparison between patients is not needed. If a change in the severity stage of a patient occurs since the last assessment with the ROSA, the clinician can perform the new assessment in the same way, using the same scenario for each item but with the new examples provided for the respective new severity stage. In such cases, the ROSA total score could not be used for a direct comparison between the new and the last assessments of the patient but the change in the severity stage itself is a global estimate for the progression of the patient disease over the past time.

Altogether, the study results reported here demonstrated that AD-relevant symptoms and their severity can be reliably assessed with the ROSA in patients at all disease severity stages. In addition to the ROSA total score, a graphical presentation of the ROSA single-item scores could easily be displayed on the ROSA sheet by connecting the ticked fields on the numerical scale. Given the possibility to use the same ROSA sheet for following assessments of a single patient, clinicians could directly compare two or more repeat evaluations based on the graphical display of the single-item scores as well. The ROSA may thus contribute to identifying particular symptoms assessed with single ROSA items that necessitate treatment and counseling. The item-specific ratings may have the advantage that, despite worsening in the ROSA total score, item scores may indicate the AD-relevant domains within the ROSA responsible for the worsening and also the domains that remain unchanged or even improve. This may be of particular benefit for studies on pharmacological and nonpharmacological interventions where effects on specific domains are expected to occur. The use of the ROSA item scores in clinical practice and research, however, needs to be appropriately validated in the future.

## Conclusions

In summary, the ROSA is a valid, reliable, and sensitive instrument for evaluating the severity of AD symptoms in daily clinical practice over time. In general, the physicians, psychologists, and nurses taking part as raters in this study have commented that the ROSA is easy to apply and useful in daily clinical practice. Nevertheless, specific training is required for all users prior to administering the ROSA for the first time. For trained raters, the administration of the ROSA takes about 15 minutes, which is much less than the time a clinician would need for administrating a set of domain-specific AD scales covering all the domains efficiently covered by the ROSA. While the sensitivity shown to change due to treatment confirms the utility of the ROSA for assessing short-term longitudinal change assessments, the broad severity range and wide range of relevant items covered by the ROSA are likely to be of great advantage for assessing change in long-term clinical trials and for assessing long-term longitudinal change.

## Abbreviations

AD: Alzheimer's disease; ADAS-Cog: Alzheimer's Disease Assessment Scale - cognitive subscale; DAD: Disability Assessment for Dementia; FAS: full analysis set; GDS: Global Deterioration Scale; ICC: intraclass correlation coefficient; MMSE: Mini-Mental State Examination; NPI: Neuropsychiatric Inventory; RI: responsiveness index; ROSA: Relevant Outcome Scale for Alzheimer's Disease; SIB: Severe Impairment Battery.

## Competing interests

In the past 5 years, VAH, SF, RI, PR, BW, and SG were members of an Advisory Board for Alzheimer's disease coordinated by Pierrel Research Europe (Essen, Germany), for which they received honorarium from Merz Pharmaceuticals (Frankfurt, Germany). KS is an employee of Merz Pharmaceuticals and receives a fixed salary. At the time of the study, FT was an employee of Merz Pharmaceuticals and received a fixed salary. RI, PR, and BW are also members of the Advisory Board at Lundbeck, in which capacity they have received consulting fees. SG is a member of the scientific advisory board, DSMB, and an investigator with Affiris, Astellas, Astra-Zeneca, BMS, Elan, Epix, ExonHit, GE Health Care, Janssen-Cilag, Lundbeck, Lilly, Merz, Myriad, Neurochem, Novartis, Pfizer, Sanofi-Aventis, Schering-Plough, Servier, Sonexa, UBC, and Wyeth. In the past 5 years, VAH has received honoraria from Bayer Healthcare, Eisai, Janssen, Lilly, Novartis, Pfizer, Servier, and Wyeth. RI has received consultant fees from APK, Austroplant, BDI, Beltz Test, BOD, Caritas Siegen, Double Helix Development, Eisai, Friedrichverlag, GE Healthcare, Hogrefe, IFE, Janssen, KDA, Landesinitiative Demenz Service NRW, LVR Düren, Medical Tribune, Med. Komm., Novartis, Pfizer, Pfrimmer Nutritia, Pierrel, Schwabe, Thieme, Urban & Vogel, Westermayer. PR has received honoraria or royalties from Eisai, Wyeth, Novartis, Janssen, and GE Healthcare.

## Authors' contributions

All authors contributed to the ROSA development and to designing of the clinical trial aiming at the ROSA validation. VAH was the coordination investigator of the clinical trial and provided critical remarks on the manuscript. FT developed the first draft of this article based on extensive discussions with all other authors. KS contributed to planning and execution of the statistical analyses. SF, RI, PR, BW, and SG provided critical remarks on the manuscript. All authors read and approved the final manuscript.
